# Supplementary material for: Influence of the Nutritional Composition of Quinoa (Chenopodium quinoa Willd.) on the Sensory Quality of Cooked Quinoa
Source: Foods. 2025 Mar 14;14(6):988. doi: 10.3390/foods14060988 (PMC11941066; doi:10.3390/foods14060988)
Supplement: Supplementary file 1 [file foods-14-00988-s001.zip › foods-3430307-supplementary.pdf]

Table S1 Sensory evaluation criteria for cooked quinoa.

Criteria	Description	Score value
Chrominance (10 points)	Normal color, obvious luster	9-10
	Normal color, slightly luster	6-8
	Abnormal color, no luster	0-5
Structure (15 points)	Uniform particle distribution and good integrity	13-15
	The particle bonding is not obvious, and most of the structures are tight and complete	8-12
	The particles stick together and explode	0-7
Flavor (20 points)	It has the unique aroma of quinoa, which is rich or light	18-20
	It has quinoa aroma, but the aroma is not obvious	15-17
	Quinoa is flavorless and odorless	8-14
	Quinoa has an unpleasant odor	0-7
Hardness (10 points)	neither too hard, nor too soft	8-10
	Feel slightly hard or soft	6-7
	Feels hard or soft	0-5
Chewiness (10 points)	The quinoa grains are Chewy	8-10
	The quinoa grains are slightly Chewy	6-7
	The quinoa grains are loose and hard, and there is residue	0-5
Viscosity (10 points)	Smooth, tacky and non-sticky	8-10
	Sticky, non-sticky teeth	6-7
	Sticky teeth or non-sticky	0-5
Taste (25 points)	Chews with a stronger, cleaner and sweeter flavor	22-25
	Chews with a light fragrance and sweetness	16-21
	Chewing, no clear flavor or sweetness, but off-flavor	0-15
Comprehensive score (100 points)	The total score is equal to the sum of the scores for each sensory indicator	
